# COVID-19 concerns among caregivers and vitamin A supplementation coverage among children aged 6–59 months in four countries in Western sub-Saharan Africa

**DOI:** 10.1017/S1368980023001258

**Published:** 2023-10

**Authors:** Melissa M Baker, Amynah Janmohamed, Djeinam Toure, Romance Dissieka, Fatou Ndiaye, Regina Khassanova, Mohamed Lamine Fofana, David Doledec

**Affiliations:** 1 Helen Keller International, P.O. Box 14195 – 00800, Nairobi, Kenya; 2 Helen Keller International, Dakar, Senegal; 3 Helen Keller International, Abidjan, Côte d’Ivoire; 4 Helen Keller International, Ouagadougou, Burkina Faso; 5 Helen Keller International, Corniche nord, Conakry, Guinea

**Keywords:** Children, COVID-19, Sub-Saharan Africa, Supplementation, Vitamin A

## Abstract

**Objective:**

To assess child vitamin A supplementation (VAS) coverage in 2019 and 2020 and explore key factors, including COVID-19 concerns, that influenced VAS status in four sub-Saharan African countries.

**Design:**

Data from eight representative household surveys were used to assess VAS coverage. Multivariable logistic regression models examined the effect of rural/urban residence, child sex and age, caregiver education, COVID-19 concern and household wealth on VAS status.

**Setting:**

Nine (2019) and 12 (2020) districts in Burkina Faso, Côte d’Ivoire, Guinea and Mali.

**Participants:**

28 283 caregivers of children aged 6–59 months.

**Results:**

Between 2019 and 2020, VAS coverage increased in Burkina Faso (82·2–93·1 %), Côte d’Ivoire (90·3–93·3 %) and Mali (76·1–79·3 %) and decreased in Guinea (86·0 % to 81·7 %). Rural children had a higher likelihood of VAS uptake compared with urban children in Burkina Faso (adjusted OR (aOR) = 4·22; 95 % CI: 3·11, 5·72), Côte d’Ivoire (aOR = 5·19; 95 % CI: 3·10, 8·70) and Mali (aOR = 1·41; 95 % CI: 1·15, 1·74). Children aged 12–59 months had a higher likelihood of VAS uptake compared with children aged 6–11 months in Côte d’Ivoire (aOR = 1·67; 95 % CI: 1·12, 2·48) and Mali (aOR = 1·74; 95 % CI: 1·34, 2·26). Moderate-to-high COVID-19 concern was associated with a lower likelihood of VAS uptake in Côte d’Ivoire (aOR = 0·55; 95 % CI: 0·37, 0·80).

**Conclusion:**

The increase in VAS coverage from 2019 to 2020 suggests that COVID-19 concerns may not have limited VAS uptake in some African countries, though geographic inequities should be considered.

Vitamin A deficiency (VAD) is of significant public health concern in areas where nutritionally poor diets inhibit adequate consumption of vital nutrients for good health. Contributing to child malnutrition, VAD poses particularly serious consequences for children under 5 years of age^(1)^. VAD remains highly prevalent in much of sub-Saharan Africa (SSA), where upwards of 50 % of children aged 6–59 months are affected^(2)^. In 2019, central, eastern and western SSA had the highest age-standardised incidence and disability-adjusted life years rates of VAD, with rates of 15 571 per 100 000 and 37 per 100 000, respectively, in western SSA^(3)^.

The WHO strongly recommends vitamin A supplementation (VAS) for infants and children of 6–59 months of age in areas where VAD is a public health problem^(4)^. Recent evidence suggests a 12 % decrease in all-cause mortality and diarrhoea-specific mortality and significant reductions in the incidence of diarrhoea, measles, Bitot’s spots, night blindness and VAD in children under 5 years of age who receive biannual VAS^(1)^. Helen Keller International and partner organisations work with governments throughout SSA to distribute VAS, mainly through semiannual door-to-door health events, and to support the integration of VAS into essential health service packages at the community level.

The global impact of COVID-19 on the health sector has been far-reaching. Widespread service disruptions have led to delayed child health events and limited outreach activities through which VAS is normally delivered^(5)^. Though reported VAS coverage in some SSA countries exceeded 80 % prior to the COVID-19 pandemic^(6)^, little is known about the effects of pandemic-related challenges and mitigation measures implemented by national governments and supporting international organisations on VAS coverage. Available data indicate that two-dose VAS coverage was substantially lower in 2020, as compared with pre-pandemic levels, in SSA and South Asia, with only approximately 40 % of targeted children reached and the lowest reported coverage in Central and West Africa^(6)^. However, these coverage estimates are based on reported country administrative data, which have shown to be unreliable when compared with other assessment methods^(7)^. This study aimed to assess VAS coverage among children aged 6–59 months in 2019 and 2020 and explore the key factors that influenced VAS uptake in 2020, including COVID-19 perceptions, in Burkina Faso, Côte d’Ivoire, Guinea and Mali.

## Methods

### Study design and population

This cross-sectional study utilised data from eight Post-Event Coverage Surveys (PECS) conducted in Burkina Faso, Côte d’Ivoire, Guinea and Mali during 2019 and 2020. All surveys were conducted within 6 weeks following door-to-door health events that included VAS distribution in each country. In 2019, PECS were conducted in each country between June and July. Due to the COVID-19 pandemic, periods of data collection varied throughout 2020, with PECS administered in July/August (Guinea), October (Mali) and December 2020/January 2021 (Burkina Faso, Côte d’Ivoire).

Each survey employed a randomised cluster design with enumeration areas selected using probability proportional to size sampling methodology. Numbers of survey strata, clusters and households varied by country in 2019 and 2020; however, the same design effect (2·5), non-response inflation factor (1·02), response rate (0·98), intracluster correlation coefficient (0·167), desired level of precision (0·95), alpha (0·05) and expected coverage (0·80) were consistent across all PECS. Survey enumeration areas in each stratum were randomly selected by the respective National Institute of Statistics in each country. Households were randomly selected from household lists in each cluster and were eligible to participate if there was a primary child caregiver present at the time of the survey and at least one child in the household was aged 6–59 months during the preceding health event. PECS were conducted by experienced and trained interviewers who were independent from the VAS distribution activities. Training tools and modalities were adapted to incorporate COVID-19 infection prevention and control measures and to sensitise interviewers on adherence to COVID-19 protocols. Additionally, survey supervisors visited 10 % of sampled households in each cluster to administer a shortened PECS to verify the data collected in the primary surveys.

Administrative systems in the study countries include a hierarchy of national, regional and local authorities. Structured according to functional and geographic responsibilities, the system includes ministries, regional authorities, and several health districts within each region that include urban and rural areas. Each country is unique in that different levels of the administrative system manage specific aspects of the VAS distribution and the PECS. In Burkina Faso, Mali and Guinea, oversight of VAS distribution and the PECS is at the regional level, whereas oversight is done at the health district level in Côte d’Ivoire. Of the 13 regions in Burkina Faso, the PECS was representative of three in 2019 and five in 2020. In Mali, five of the 11 regions were represented in 2019 and 2020. Of the 113 health districts in Côte d’Ivoire, the PECS was representative of 71 health districts in 2019 and 72 health districts in 2020. In Guinea, the PECS was nationally representative in 2019 and 2020. In all countries, both the 2019 and 2020 PECS included questions on: household socio-demographic characteristics; health-seeking practices; health event/campaign communication methods; vitamin A and VAS knowledge and attitudes; VAS status; and reasons for non-supplementation. An additional module in the 2020 PECS included questions related to COVID-19 knowledge, attitudes and behaviours, as well as compliance with infection control measures.

### Statistical analysis

Survey data were electronically recorded using smartphones and directly exported to Excel for preliminary analysis. Sampling weights were calculated to generate representative estimates for respective survey regions/districts. SPSS Complex Samples 26.0 (IBM Corp) was used to conduct descriptive, bivariate and multivariable analyses. The study outcome was child VAS status, as determined from the child’s health card or caregiver recall. Based on these data, VAS coverage estimates for 2019 and 2020 were calculated for each country. Logistic regression models examined the effects of rurality, child sex and age, caregiver education, household wealth and level of COVID-19 concern on VAS status. Model variables were included based on a significant (*P* < 0·05) bivariate relationship and/or acknowledged association with VAS. Results are presented as OR with 95 % CI (*α* = 0·05). Sample-weighted estimates are presented for all outcome results. Separate analyses were conducted for each country. Ethical approval was obtained from the respective institutions in each country, and informed consent was required from all survey respondents prior to participation.

## Results

Data were collected from a total of *n* 28 283 respondents across the eight surveys. The proportion of rural *v*. urban households varied across countries, with approximately 80 % and 70 % of surveyed households in rural localities in Burkina Faso and Mali, respectively, and less than 50 % of households in rural areas in Côte d’Ivoire and Guinea (Table 1). In all countries, the majority of respondents in the 2019 and 2020 PECS were mothers. The largest proportions of fathers, grandparents and other respondents were in Guinea in the 2019 (41·1 %) and 2020 (47·7 %) surveys. In Côte d’Ivoire and Mali, there was an approximate 10 % decrease in the percentage of respondents who were mothers between 2019 and 2020. In all countries, more than 90 % of mothers in the household were aged 20 years or older. The majority of mothers reported having no formal education, with the highest percentage in Burkina Faso (73·1 % in 2019; 72·6 % in 2020). Infants aged 6–11 months comprised 9–13 % of surveyed children and child sex parity was observed across all four countries, with the largest difference (52·6 % male *v*. 47·4 % female) in Côte d’Ivoire in 2020 (Table 1). In 2020, 40·4 % of households in Burkina Faso, 42·7 % in Côte d’Ivoire, 39·9 % in Guinea and 54·5 % in Mali were classified as having very poor or poor wealth status. In the 2020 surveys, the majority of respondents in all countries expressed concern about COVID-19. Approximately one in five respondents in Côte d’Ivoire, Guinea and Mali and almost one-quarter (24·1 %) in Burkina Faso reported being very concerned about COVID-19, while 8·3 % of respondents in Burkina Faso, 11·2 % in Côte d’Ivoire, 15·7 % in Guinea and 10·8 % in Mali reporting not being concerned (Table 2). Notwithstanding the level of COVID-19 concern, more than 90 % of respondents in all countries reported they were willing for their child(ren) to receive VAS at the household (Table 2).

**Table 1:** Participant characteristics

	Burkina Faso				Côte d’Ivoire				Guinea				Mali			
	2019		2020		2019		2020		2019		2020		2019		2020	
	*n* 3432		*n* 5587		*n* 4647		*n* 3346		*n* 2526		*n* 2727		*n* 2221		*n* 3797	
	*n*	%	*n*	%	*n*	%	*n*	%	*n*	%	*n*	%	*n*	%	*n*	%
Relationship to child																
Mother	2661	77·6	4061	72·9	3396	73·1	2094	62·6	1486	58·8	1426	52·3	1678	75·6	2407	63·4
Father	297	8·7	462	8·3	568	12·2	533	15·9	347	13·7	562	20·6	163	7·3	688	18·1
Grandparent	203	5·9	440	7·9	377	8·1	324	9·7	197	7·8	294	10·8	123	5·5	369	9·7
Other	270	7·9	605	10·9	306	6·6	395	11·8	496	19·6	445	16·3	257	11·6	333	8·8
Mother’s age																
15–19 years	68	2·6	142	3·5	98	3·1	64	3·2	98	7·4	74	5·2	91	5·4	91	3·8
20–24 years	556	20·9	811	20·0	626	19·7	341	17·0	246	18·7	290	20·3	383	22·8	436	18·1
25–29 years	720	27·1	1004	24·7	791	24·9	485	24·2	339	25·8	346	24·3	463	27·6	569	23·6
30–34 years	602	22·6	963	23·7	727	22·9	478	23·9	211	16·0	230	16·1	340	20·3	506	21·0
>= 35 years	676	25·4	990	24·4	933	29·4	636	31·7	421	32·0	261	18·3	359	21·4	652	27·1
Mother’s educational status																
None	1945	73·1	2950	72·6	2083	61·4	1095	52·4	627	42·5	876	61·4	1045	62·3	1658	68·9
Primary	382	14·4	671	16·5	807	23·8	550	26·3	238	16·1	165	11·6	424	25·3	415	17·2
Secondary or higher	334	12·6	440	10·8	502	14·8	446	21·4	460	31·2	267	18·7	209	12·5	334	13·9
Sex of child																
Male	1675	51·6	2802	50·3	2322	50·0	1759	52·6	1292	51·1	1399	51·3	1133	51·0	1961	51·6
Female	1569	48·4	2766	49·7	2325	50·0	1587	47·4	1234	48·9	1328	48·7	1088	49·0	1836	48·4
Age of child																
6–11 months	416	12·8	575	10·3	602	13·0	389	11·6	296	11·7	252	9·2	239	10·8	351	9·2
12–23 months	756	23·3	1255	22·5	1246	26·8	839	25·1	490	19·4	579	21·2	488	22·0	866	22·8
24–35 months	698	21·5	1309	23·5	1085	23·3	799	23·9	583	23·1	657	24·1	510	23·0	884	23·3
36–47 months	660	20·3	1204	21·6	1079	23·2	760	22·7	565	22·4	622	22·8	536	24·1	890	23·4
48–59 months	714	22·0	1225	22·0	635	13·7	559	16·7	592	23·4	617	22·6	448	20·2	806	21·2
Area of residence																
Rural	2715	79·1	4433	79·3	2160	46·5	1203	36·0	850	33·7	1316	48·3	1510	68·0	2852	75·1
Urban	691	20·1	1154	20·7	2487	53·5	2143	64·0	1676	66·3	1411	51·7	711	32·0	945	24·9

**Table 2:** Level of COVID-19 concern and attitudes towards vitamin A supplementation among caregivers of children aged 6–59 months

	Burkina Faso		Côte d’Ivoire		Guinea		Mali	
	*n* 5177		*n* 3072		*n* 2727		*n* 3797	
	%	CI	%	CI	%	CI	%	CI
Level of concern about COVID-19								
Not concerned	8·3	7·5, 9·2	11·2	10·2, 12·4	15·7	14·2, 17·5	10·8	9·8, 11·8
A bit concerned	20·3	18·9, 21·9	16·1	14·9, 17·5	18·6	17·0, 20·4	11·5	10·5, 12·6
Concerned	47·3	45·5, 49·1	53·3	51·5, 55·0	45·6	43·5, 47·8	57·1	55·5, 58·7
Very concerned	24·1	22·6, 25·7	19·4	18·0, 20·8	20·0	18·3, 21·8	20·6	19·3, 21·9
Willingness for child to receive VAS at home								
Yes	93·8	92·8, 94·6	91·7	90·7, 92·7	95·9	95·0, 96·7	93·5	92·6, 94·3
No	6·2	5·4, 7·2	8·3	7·3, 9·3	4·1	3·3, 5·0	6·5	5·7, 7·4

In 2019, VAS coverage among children aged 6–59 months ranged from 76·1 % in Mali to 90·3 % in Côte d’Ivoire (Table 3). VAS coverage was higher in 2020, as compared with 2019, in Burkina Faso (93·1 % *v*. 82·2 %), Côte d’Ivoire (93·3 % *v*. 90·3 %) and Mali (79·3 % *v*. 76·1 %). In Guinea, a 4·3 % decrease in VAS coverage was observed between 2019 (86·0 %) and 2020 (81·7 %) (Table 3). In 2020, VAS coverage in the four countries was significantly higher among children in rural households, as compared with those in urban households (Burkina Faso: 93·4 % *v*. 81·0 %, *P* < 0·001; Côte d’Ivoire: 98·2 % *v*. 90·5 %, *P* < 0·001; Guinea: 85·9 % *v*. 64·8 %, *P* < 0·001; Mali: 81·3 % *v*. 72·1 %, *P* < 0·001) and was higher among children aged 24–59 months, as compared with those aged 6–11 months in Guinea (83·3 % *v*. 71·1 %, *P* < 0·001) and Mali (80·7 % *v*. 71·0 %, *P* < 0·001). No differences in VAS coverage were observed between male and female children in any of the four countries. VAS coverage was lowest among children in the wealthiest household category in Burkina Faso (*P* = 0·026), Côte d’Ivoire (*P* < 0·001), Guinea (*P* < 0·001) and Mali (*P* < 0·001) and was lowest among children of caregivers with the highest level of education in Burkina Faso (*P* = 0·028), Côte d’Ivoire (*P* = 0·036) and Mali (*P* < 0·001). Among children in households reporting a high level of COVID-19 concern, VAS coverage was 91·9 % in Burkina Faso, 91·5 % in Côte d’Ivoire, 85·9 % in Guinea and 78·1 % in Mali. In Mali, VAS coverage was 9·3 % higher (78·1 % *v*. 68·8 %, *P* = 0·029) for children in highly concerned households, as compared with not concerned households. Approximately 10 % more caregivers in rural, as compared with urban, households reported willingness for their child(ren) to receive VAS at the household in Burkina Faso, Côte d’Ivoire and Mali, whereas this difference was approximately 20 % in Guinea.

**Table 3:** VAS coverage among children aged 6–59 months during 2019 and 2020 campaigns

	2019		2020	
	%	CI	%	CI
Burkina Faso	82·2	80·8, 83·6	93·1	92·2, 93·9
Côte d’Ivoire	90·3	88·7, 91·7	93·3	92·4, 94·1
Guinea	86·0	84·3, 87·6	81·7	80·0, 83·2
Mali	76·1	74·2, 78·0	79·3	77·9, 80·6

In Burkina Faso, Côte d’Ivoire and Mali, the most frequently reported reason for children not receiving VAS during both the 2019 and 2020 events was health workers not coming to the household to provide VAS (Table 4). In Guinea, the child being absent at the time of supplementation (41·2 %) and health workers not coming to administer VAS at the household (55·7 %) were the most commonly mentioned barriers to supplementation during the 2019 and 2020 events, respectively. In 2020, the child not being present at the time of VAS distribution was reported by approximately 30 % of respondents in Côte d’Ivoire. In Mali, about 20 % of respondents reported not being informed about VAS and/or the health event, while in Burkina Faso 12–15 % of respondents cited a lack of VAS supply as the reason for non-supplementation. VAS refusal ranged from < 0·5 % of households in Burkina Faso in 2019 and 2020 to 13·6 % of households in the 2019 Guinea survey (Table 4). Mask wearing by health workers distributing VAS at the household during the 2020 events was observed by 86·9 % of respondents in Burkina Faso, 84·5 % in Côte d’Ivoire, 72·6 % in Guinea and 67·0 % in Mali.

**Table 4:** Reasons for children aged 6–59 months not receiving vitamin A supplementation during 2019 and 2020 campaigns

	Burkina Faso				Côte d’Ivoire				Guinea				Mali			
	2019 (*n* 563)		2020 (*n* 481)		2019 (*n* 310)		2020 (*n* 225)		2019 (*n* 382)		2020 (*n* 647)		2019 (*n* 516)		2020 (*n* 781)	
	%	CI	%	CI	%	CI	%	CI	%	CI	%	CI	%	CI	%	CI
Reason for non-VAS																
Child absent	13·9	11·2, 17·1	21·8	17·0, 27·6	18·7	13·1, 25·9	29·9	24·2, 36·3	41·2	34·8, 47·9	22·2	18·4, 26·5	16·6	13·4, 20·3	9·9	8·0, 12·3
Child sick	0·2	0·0, 0·7	1·1	0·3, 4·0	3·3	0·7, 13·3	0·4	0·1, 3·0	3·0	1·2, 7·4	0·1	0·0, 0·6	0·8	0·3, 2·1	0·4	0·1, 1·4
Health workers did not come to house	67·0	62·9, 70·9	44·5	38·5, 50·7	73·9	65·1, 81·2	56·3	49·6, 62·7	27·8	22·5, 33·8	55·7	50·9, 60·5	54·4	49·9, 58·8	67·7	64·2, 71·0
No VAS supply	14·5	11·7, 17·8	12·4	7·9, 19·0	N/A		N/A		0·5	0·1, 1·7	1·2	0·4, 3·1	4·2	2·8, 6·4	0·8	0·4, 1·8
Not informed	6·5	4·7, 9·0	16·5	12·0, 22·3	0·4	0·1, 1·6	6·4	3·9, 10·4	6·9	4·5, 10·4	3·2	1·8, 5·5	20·4	17·1, 24·1	17·4	14·8, 20·5
Refused	0·2	0·0, 1·3	0·2	0·1, 0·9	0·7	0·2, 2·5	5·9	3·4, 9·9	13·6	9·7, 18·6	6·5	4·5, 9·4	1·9	1·0, 3·8	3·0	1·9, 4·7

N/A: Data not collected.

As we could not statistically compare 2019 and 2020 coverage estimates due to country sampling variations, the following findings were generated from adjusted models that included data from the 2020 surveys. Children in rural areas had a higher likelihood of receiving VAS, as compared with those in urban areas, in Burkina Faso (OR = 4·22; 95 % CI: 3·11, 5·72), Côte d’Ivoire (OR = 5·19; 95 % CI: 3·10, 8·70), Guinea (OR = 2·94; 95 % CI: 2·30, 3·77) and Mali (OR = 1·41; 95 % CI: 1·15, 1·74) (Table 5). Children aged 12–59 months were more likely to receive VAS, as compared with those aged 6–11 months, in Côte d’Ivoire (OR = 1·67; 95 % CI: 1·12, 2·48), Guinea (OR = 1·93; 95 % CI: 1·34, 2·77) and Mali (OR = 1·74; 95 % CI: 1·34, 2·26). In Mali, caregiver education was associated with a higher likelihood of child VAS (OR = 1·26; 95 % CI: 1·04, 1·53), while children in the middle household wealth (OR = 0·45; 95 % CI: 0·37, 0·54) and highest wealth (OR = 0·37; 95 % CI: 0·30, 0·46) categories had a lower likelihood of VAS. A moderate to high level of caregiver concern regarding COVID-19 was associated with a lower likelihood of child VAS only in Côte d’Ivoire in both the unadjusted (OR = 0·50; 95 % CI: 0·34, 0·72) and multivariable (adjusted OR (aOR) = 0·55; 95 % CI: 0·37, 0·80) models (Table 5). A further exploration of COVID-19 concerns by socio-economic position in Côte d’Ivoire revealed that middle (OR = 1·40; 95 % CI: 1·12, 1·74) and highest (OR = 1·66; 95 % CI: 1·38, 1·98) wealth households were more likely to have a moderate/high level of COVID-19 concern, as compared with the lowest wealth households. In addition, rural households were less likely to have a moderate/high level of COVID-19 concern, as compared with urban households (OR = 0·75; 95 % CI: 0·64, 0·88).

**Table 5:** Multivariable regression associations between predictor variables and vitamin A supplementation among children aged 6–59 months during 2020 campaigns

	Burkina Faso		Côte d’Ivoire		Guinea		Mali	
	AOR	CI	AOR	CI	AOR	CI	AOR	CI
Concern about COVID-19								
No/low (ref)	–	–	–	–	–	–	–	–
Moderate/high*	1·15	0·85, 1·54	0·55	0·37, 0·80	1·04	0·82, 1·33	1·12	0·91, 1·38
Area of residence								
Urban (ref)	–	–	–	–	–	–	–	–
Rural	4·22	3·11, 5·72	5·19	3·10, 8·70	2·94	2·30, 3·77	1·41	1·15, 1·74
Sex of child								
Male (ref)	–	–	–	–	–	–	–	–
Female	1·00	0·77, 1·32	0·92	0·69, 1·23	0·89	0·71, 1·11	0·95	0·80, 1·12
Age of child								
6–11 months (ref)	–	–	–	–	–	–	–	–
12–59 months	1·39	0·94, 2·06	1·67	1·12, 2·48	1·93	1·34, 2·77	1·74	1·34, 2·26
Caregiver education								
None (ref)	–	–	–	–	–	–	–	–
Any	1·27	0·92, 1·76	0·97	0·73, 1·29	1·12	0·88, 1·42	1·26	1·04, 1·53
Wealth category								
Lowest (ref)	–	–	–	–	–	–	–	–
Middle	0·80	0·56, 1·14	0·94	0·61, 1·45	1·00	0·74, 1·37	0·45	0·37, 0·54
Highest	0·99	0·72, 1·36	0·95	0·66, 1·37	0·77	0·56, 1·05	0·37	0·30, 0·46

AOR, adjusted OR.

* Unadjusted estimates: Burkina Faso (OR = 1·13; 95 % CI: 0·84, 1·51); Côte d’Ivoire (OR = 0·50; 95 % CI: 0·34, 0·72); Guinea (OR = 0·93; 95 % CI: 0·74, 1·18); and Mali (OR = 1·02; 95 % CI: 0·84, 1·25).

## Discussion

We sought to assess VAS coverage and examine the influence of key factors, including COVID-19 concerns, on VAS uptake among children aged 6–59 months in four countries in SSA. The higher VAS coverage observed in 2020, as compared with pre-pandemic levels, in three countries was unexpected. Global evidence (based on administrative data) from the same time period suggests the suspension of door-to-door (mass campaign) health events caused a 19 % decrease in two-dose VAS coverage in 2020, as compared with 2019, particularly during the first half of the year which subsequently impacted two-dose VAS coverage in 2020^(8)^. Despite the challenges imposed by the pandemic, Helen Keller International managed to promote, facilitate and support the distribution of VAS through two rounds of door-to-door health events within most supported countries in 2020, albeit while respecting the mitigation measures recommended by WHO and country governments. Standard supplementation procedures were followed in Helen Keller International-supported programmes in Burkina Faso, Côte d’Ivoire and Mali during the pandemic, with the addition of COVID-19 infection control measures. In Guinea, VAS distribution modalities changed during the first campaign round in 2020 from a pre-pandemic 5-d distribution event to a multi-phased approach targeting different districts over a 2-month period. This altered delivery strategy may have contributed to the lower VAS coverage observed in Guinea in 2020, as compared with 2019. Furthermore, in Guinea, a higher percentage of survey respondents were non-mothers which could have affected knowledge and or recall of children’s supplementation status.

A possible explanation for the higher-than-expected VAS coverage observed during the pandemic in our study is apprehensions surrounding COVID-19 did not negatively impact VAS uptake in areas supported by Helen Keller International where VAS distribution occurred. There may have been increased propensity towards positive health-seeking behaviours due to heightened health and safety awareness from media and other sources, in addition to intensive mobilisation efforts by Helen Keller International and partners that incorporated strategies to sensitise communities and adopt infection prevention and control measures during door-to-door visits^(9)^. This is further supported by the vast majority (83–94 %) of respondents affirming they could reduce COVID-19 transmission through their own actions. In addition to community knowledge about vitamin A and its importance for child health, the likely greater attention to hygiene/disease prevention during the pandemic may have bolstered parental willingness for VAS to protect children from illness at a time when access to routine health services was especially limited. Furthermore, across the four countries, high VAS coverage (78–92 %) was observed among households reporting a high level of concern about COVID-19. Though this was unexpected, as 65–78 % of households expressed being concerned or very concerned about COVID-19, these findings further support the likelihood that pandemic-related fears did not greatly affect perceptions and attitudes towards VAS in these settings.

Though the precise reasons for the lower VAS coverage observed in Guinea in 2020 are not known, the large proportion of caregivers who reported that their child(ren) did not receive VAS due to health workers not coming to their community suggests that logistical constraints, perhaps worsened by pandemic-related challenges, may have played a greater role in Guinea. Notwithstanding, the lack of distribution in target areas was also the primary reported barrier to VAS receipt in Burkina Faso, Côte d’Ivoire and Mali and reduced accessibility due to geographic, pandemic and/or other factors presumably affected VAS delivery in all countries. Though community outreach challenges were likely exacerbated during the pandemic, our findings are consistent with results from other studies conducted prior to the pandemic indicating that households not being reached during door-to-door campaigns is a key contributor to missed VAS doses in the SSA context^(10,11)^.

In 2020, VAS coverage was > 90 % in Burkina Faso and Côte d’Ivoire and approximately 80 % in Guinea and Mali. Though these levels meet the WHO/UNICEF guidelines for at least 80 % coverage as a measure of effective VAS programming, subnational disparities and socio-economic inequities remain, particularly between rural and urban areas. In 2020, VAS coverage was 10–20 % higher in rural, as compared with urban, areas across the four countries. A possible reason for this disparity may be poorer accessibility in densely populated urban areas that was exacerbated by pandemic-related disruptions in health services. Apart from the unique challenges imposed by COVID-19, mixed evidence exists for the association between rural/urban residence and VAS, with both higher rural and urban coverage observed in different settings^(11–18)^.

In our study, a higher level of caregiver education and increased household wealth were not predictors of VAS uptake in Burkina Faso, Côte d’Ivoire and Guinea, while caregiver education was positively associated with VAS in Mali. Although these were unexpected findings, it is possible that caregivers with a higher level of education did not consider VAS to be necessary, whereas households with a lower socio-economic status may have been more willing for their children to receive VAS due to increased perceptions of vulnerability to illness, particularly during the pandemic. Additionally, heightened COVID-19 concern among more educated individuals could have influenced decisions surrounding child VAS at the household, as evident in Côte d’Ivoire where wealthier urban households had increased apprehensions about COVID-19, as compared with poorer rural households.

Therefore, our findings suggest good VAS compliance across socio-economic strata, also possibly due to effective community sensitisation strategies. While our findings contrast with available evidence from SSA indicating higher VAS uptake among advantaged children with educated mothers and wealthier households^(12,14–21)^, large multi-country studies tend to pool data from various delivery models, thereby potentially masking key contributors to VAS uptake. We hypothesise that education and wealth status may not be key determinants of VAS coverage in door-to-door delivery models, but rather more significant factors in facility-based programmes that require active seeking of health services. In a pooled analysis of 44 PECS conducted in 13 SSA countries, caregivers with some formal education were more likely to have brought their children to a fixed site for VAS and less likely to have their children receive VAS during door-to-door distribution events^(11)^.

In Côte d’Ivoire, Guinea and Mali, children aged 12 months and older had a higher likelihood of VAS, as compared with infants 6–11 months of age. Similarly, a pooled analysis of Demographic and Health Surveys conducted from 2011 to 2015 in 23 SSA countries showed children aged 12–23 months (OR = 1·63; 95 % CI: 1·58, 1·69) and 24–35 months (OR = 1·38; 95 % CI: 1·34, 1·43) were more likely to receive VAS, as compared with those aged 6–11 months^(14)^. The lower VAS coverage observed for children under one year of age in our study could indicate challenges reaching these youngest children during the campaigns and/or that these infants may have been provided VAS during their routine childhood immunisation visits in the months preceding the survey.

A key strength of our study was the inclusion of data representative at the regional/district level from four countries in the West African region. In addition, VAS distribution followed a similar door-to-door delivery model in all countries, and standard survey design, sampling and data collection methods were employed in all regions. The 6-week time interval between VAS distributions and the post-event household surveys likely minimised the effect of recall bias. As PECS are cross-sectional, we examined associations between VAS and explanatory variables but could not infer causality. Though we controlled for potential confounding variables, there may have been influences from other factors not accounted for in the study. We could not statistically compare 2019 and 2020 coverage estimates due to country sampling variations. Moreover, the results should not be compared across countries as the individual surveys were not designed to detect inter-country differences. The time of year during which VAS distribution occurred varied within and across countries, which could have affected VAS delivery mechanisms and coverage. The limited information available in our study regarding disruptions in service delivery due to COVID-19 that could have affected VAS coverage in our settings should be considered. In addition, as the PECS assessed door-to-door VAS coverage, we were not able to capture VAS doses administered at health facilities or other fixed sites. Lastly, our findings should be considered with the caveat that surveys and administrative records serve distinct purposes with different populations, sampling, data collection methods and outcome measures (single- *v*. two-dose) and, therefore, tend to produce dissimilar VAS coverage estimates.

In our study, COVID-19-related concerns were not limiting factors to the uptake of VAS through house-to-house delivery in the West African context, suggesting high coverage can be achieved during a public health crisis. Nonetheless, geographic disparities in coverage, particularly between urban and rural areas, should be further investigated and considered in the planning and implementation of door-to-door VAS distribution. This is especially important during public health crises when service disruptions further exacerbate health disparities and prevent equitable coverage in certain areas. Targeted post-pandemic efforts are needed for missed VAS doses, ideally provided with other essential child health services, including immunisations, deworming and screening for acute malnutrition. Achieving substantial reductions in child mortality requires children aged 6–59 months to receive twice-yearly vitamin A supplements where VAD is a public health problem, such as in these study settings. Though our findings suggest high coverage can be achieved during a public health crisis, local factors that may have affected programme implementation during the pandemic should be considered, along with targeted efforts for hard-to-reach areas.
